# Complete Genome Sequences of Two Salmonella enterica Lytic Phages, NBSal006 and NBSal007

**DOI:** 10.1128/MRA.00969-20

**Published:** 2020-10-01

**Authors:** Carlos D. Llanos, Renzo Punil, Enrique Mamani, Carlo Mormontoy, Jorge A. Bardales

**Affiliations:** a Nextbiotics, Inc., Oakland, California, USA; b Departamento de Microbiología y Parasitología, Universidad Nacional Mayor de San Marcos, Lima, Peru; DOE Joint Genome Institute

## Abstract

The use of bacteriophages as antimicrobial agents represents a promising alternative for the control of pathogenic bacteria. Here, we present the complete genome sequences of two novel Salmonella enterica lytic bacteriophages, NBSal006 and NBSal007, candidates for *Salmonella* biocontrol.

## ANNOUNCEMENT

Salmonella enterica is a Gram-negative pathogen recognized as one of the most common causal agents of foodborne infections (1, 2). Poultry flocks are one of the main reservoirs of *Salmonella* and thus are an ideal target for its control using bacteriophage-based antimicrobials. An initial step that is necessary for the development of such phage-based antimicrobials is the rigorous characterization of novel phages with strong lytic activity. Here, we describe the isolation and characterization of two novel *Salmonella* bacteriophages, NBSal006 and NBSal007.

To isolate bacteriophages, 50 g of waste samples was collected from Peruvian poultry (location, 11°58′22.1ʺS, 77°06′17.0ʺW); these samples were stored at 4°C until their use in phage isolation. For phage isolation, the soft agar overlay method was performed as described by Adams (3), using Salmonella enterica AR-404 (CDC and FDA Antibiotic Resistance Isolate Bank) as the host; 3 passages were required to identify single plaques. Isolated phages were amplified by incubating phage and *Salmonella* cultures for 18 h at 37°C in tryptic soy broth or agar.

Phage genomic DNA was extracted using a modified phenol-chloroform method, including a DNase step to limit bacterial DNA contamination (4). The QIAseq FX single-cell DNA library kit was used to prepare the genomic library, followed by sequencing on the MiSeq Illumina platform (151-bp paired-end reads). Raw reads were trimmed using Fastp (5) and assembled using Unicycler (6), followed by the SPAdes genome assembler v3.9.0 using the Unicycler contigs as trusted contigs to obtain the complete genome sequence for each phage (7). Phage genome completeness was checked using the PHASTER tool, for which the completeness score is based on the proportion of phage genes in the genome (8); also, it was corroborated by identifying the genome ends and packing strategy using the PhageTerm tool (9). Genomic annotation was performed using the RAST server (10), and the tRNA presence was checked with tRNAscan-SE v2.0, included in the RAST server (11). Default parameters were used for all software unless otherwise specified.

The numbers of sequencing reads generated for NBSal006 and NBSal007 were 5,008,144 and 5,111,521, respectively; phage NBSal006 had a genome of 41,850 bp with 3,915× coverage, and NBSal007 had a genome of 43,747 bp with 4,241× coverage. Both phage genomes had a G+C content of 49.8%. The genomic annotation for the phages presented 56 coding sequences with assigned function out of 66 annotated coding sequences for NBSal006 and 54 out of 57 for NBSal007. Moreover, no tRNA coding sequences were identified in the phages. Both phages presented circularly permutated genomes with redundant ends and a headful packaging mechanism. The genomic relation to other phages was evaluated with a nucleotide BLAST search, which showed notable similarity across the entire phage genome with phages from the *Siphoviridae* family, particularly NBSal006 with the previously described phage Shelanagig (nucleotide identity, 95.67%) and phage NBSal007 with the phage vB_StyS-sam (nucleotide identity, 92.03%) Table 1.

**TABLE 1:** Characteristics of phages NBSal006 and NBSal007

Phage name (GenBank accession no.)	No. of paired-end reads (million)	Genome length (bp)	Coverage (×)	Packaging strategy^*a*^	No. of genes	No. of genes with predicted functions	G+C content (%)	Closest phage (GenBank accession no., % identity)
NBSal006 (MT677933)	5	41,850	3,915	Headful (Pac)	66	56	49.8	Shelanagig (MK931446.1, 95.67)
NBSal007 (MT677934)	5.1	43,747	4,241	Headful (Pac)	57	54	49.8	vB_StyS-sam (LC507823.1, 92.03)

a Pac, subtype of the genome packaging mechanism used by the phage (8).

The discovery of new phages with strong lytic activity is an essential step in the development of phage-based antimicrobial products. The study of the new phages’ genomes enables us to have a better understanding of their biology and have a solid foundation to conduct further functional studies on them. Here, we present the complete genome sequences of 2 Salmonella enterica lytic phages with the potential to be used in the development of a phage-based antimicrobial for the biocontrol of Salmonella enterica in poultry.

### Data availability.

The genome sequences and raw data have been deposited in GenBank under the accession numbers MT677933 and SRR12133959 (NBSal006) and MT677934 and SRR12127194 (NBSal007).
